# Demonstration of highly efficient dual gRNA CRISPR/Cas9 editing of the homeologous *GmFAD2–1A* and *GmFAD2–1B* genes to yield a high oleic, low linoleic and α-linolenic acid phenotype in soybean

**DOI:** 10.1186/s12870-019-1906-8

**Published:** 2019-07-15

**Authors:** Phat T. Do, Cuong X. Nguyen, Hien T. Bui, Ly T. N. Tran, Gary Stacey, Jason D. Gillman, Zhanyuan J. Zhang, Minviluz G. Stacey

**Affiliations:** 1 Division of Plant Sciences, University of Missouri, Columbia, MO 65211 USA; 2 Present address: Institute of Biotechnology, Vietnam Academy of Science and Technology, Hanoi, Vietnam; 3 Plant Biotechnology Innovation Laboratory, Division of Plant Sciences, University of Missouri, Columbia, MO 65211 USA; 4 Division of Biochemistry, University of Missouri, Columbia, MO 65211 USA; 5 USDA-ARS, University of Missouri, Columbia, MO 65211 USA

**Keywords:** GmFAD2, Oleic acid, Linoleic acid, CRSIPR/Cas9, Soybean

## Abstract

**Background:**

CRISPR/Cas9 gene editing is now revolutionizing the ability to effectively modify plant genomes in the absence of efficient homologous recombination mechanisms that exist in other organisms. However, soybean is allotetraploid and is commonly viewed as difficult and inefficient to transform. In this study, we demonstrate the utility of CRISPR/Cas9 gene editing in soybean at relatively high efficiency. This was shown by specifically targeting the Fatty Acid Desaturase 2 (GmFAD2) that converts the monounsaturated oleic acid (C18:1) to the polyunsaturated linoleic acid (C18:2), therefore, regulating the content of monounsaturated fats in soybean seeds.

**Results:**

We designed two gRNAs to guide Cas9 to simultaneously cleave two sites, spaced 1Kb apart, within the second exons of *GmFAD2–1A* and *GmFAD2–1B.* In order to test whether the Cas9 and gRNAs would perform properly in transgenic soybean plants, we first tested the CRISPR construct we developed by transient hairy root transformation using *Agrobacterium rhizogenesis* strain K599. Once confirmed, we performed stable soybean transformation and characterized ten, randomly selected T0 events. Genotyping of CRISPR/Cas9 T0 transgenic lines detected a variety of mutations including large and small DNA deletions, insertions and inversions in the *GmFAD2* genes. We detected CRISPR- edited DNA in all the tested T0 plants and 77.8% of the events transmitted the *GmFAD2* mutant alleles to T1 progenies. More importantly, null mutants for both *GmFAD2* genes were obtained in 40% of the T0 plants we genotyped. The fatty acid profile analysis of T1 seeds derived from CRISPR-edited plants homozygous for both *GmFAD2* gene*s* showed dramatic increases in oleic acid content to over 80%, whereas linoleic acid decreased to 1.3–1.7%. In addition, transgene-free high oleic soybean homozygous genotypes were created as early as the T1 generation.

**Conclusions:**

Overall, our data showed that dual gRNA CRISPR/Cas9 system offers a rapid and highly efficient method to simultaneously edit homeologous soybean genes, which can greatly facilitate breeding and gene discovery in this important crop plant.

**Electronic supplementary material:**

The online version of this article (10.1186/s12870-019-1906-8) contains supplementary material, which is available to authorized users.

## Background

Soybean [*Glycine max* (L.) Merr.] is a major oil and protein crop grown worldwide which accounts for approximately 53–56% of USA vegetable oil consumption (www.soystats.com, accessed 07-17-2018). Soybean oil is used extensively in the food industry for cooking, baking and frying, but is also utilized for biodiesel production and industrial applications [1]. Soybean seed oil from a typical representative sample includes five major fatty acids: 11% palmitic (16:0), 4% stearic (18:0), 25% oleic (18:1), 52% linoleic (18:2) and 8% linolenic (18:3) [2]. The relatively high concentration of linoleic and α-linolenic acids (~ 60–70%) causes oxidative instability in the quality of soybean oil and soybean-derived processed foods. In addition, the lower content of monounsaturated fatty acids in soybean seeds makes soybean oil less competitive to other oils such as canola and olive oils. The conventional approach to overcome these constraints has been to partially hydrogenate soybean oil chemically, which not only adds to the cost but also generates *trans* fats, a significant health concern [3]. Moreover, in 2015, U.S. food and drug administration announced that partially hydrogenated oils are not generally recognized as safe (https://www.fda.gov/food/). Therefore, conventional breeding and genetic engineering approaches have been used to enrich the content of oleic acid and reduce levels of linoleic and α-linolenic acids in soybean seeds [2, 4]. The delta-12 Fatty Acid Desaturase 2 (FAD2) enzyme catalyzes the conversion of oleic acid to linoleic acid, which is further converted to α-linolenic acids by the action of delta-9 Fatty Acid Desaturase 3 (FAD3) enzymes [5, 6]. Loss of enzyme function reduces the relative amounts of both linoleic and α-linolenic acids simultaneous with greater accumulation of oleic acid, an ideal fatty acid composition for cooking and frying. The soybean genome encodes multiple copies of *GmFAD2* genes, of which the *GmFAD2–1A* and *GmFAD2–1B* genes have been shown to play an important role in controlling the level of oleic acid due to their high expression in developing seeds [6]. *GmFAD2–1A* and *GmFAD2–1B* share 99% coding sequence identity and are located in paralogous regions of chromosomes 10 and 20, respectively.

Previous research showed that RNAi silencing to reduce *GmFAD2* expression in soybean increased seed oleic acid content [7–9]. As would be expected, soybean cultivars harboring homozygous mutations in both *GmFAD2–1A* and *GmFAD2–1B* also showed significant increases in oleic acid with a concomitant reduction of linoleic and linolenic fatty acids. Nevertheless, no high oleic phenotype was observed with the *GmFAD2–1B* mutant alone [10, 11]. In addition, the large deletions of soybean *GmFAD2–1* genes in some of the mutant lines was found to adversely affect the stability of oleic acid content and seed yield [11, 12]. Additional mutants in both *GmFAD2–1A* and *GmFAD2–1B* were induced by gene editing using transcription activator like effector nucleases (TALENS). However, the transmission of mutations of both *GmFAD2* genes to progeny lines was low [13]. Moreover, the wide use of this technology has been hampered by challenges in engineering with this system.

Recently, CRISPR/Cas9 [clustered, regularly interspaced, short palindromic repeats (CRISPR)/CRISPR-associated 9 (Cas9)] systems have been engineered to create efficient genome modifications in multiple eukaryotic systems. The use of CRISPR/Cas9 has expanded rapidly and successfully applied in various plant species to induce targeted genome editing [14–16]. Stable transformation of soybean has traditionally been considered difficult relative to other plant species, with efficiencies commonly reported below 10%. Hence, early studies achieved successful CRISPR/Cas9-targeted mutagenesis using culture or transient transformation, such as hairy roots, protoplasts, embryogenic callus, and whole plants derived from either *Agrobacterium* or bombardment-mediated transformation systems [17–23]. More recently, isolated reports have appeared demonstrating stable transgenic mutant soybean derived from CRISPR/Cas9 editing with a single guide RNA [19, 23–25]. The highest reported editing efficiency in stable transgenic soybean was 76% [19]. Furthermore, similar to the editing results from other crops, most CRISPR/Cas9 induced mutations in soybean are present as small insertions or deletions, which were detected by restriction enzyme digestion-suppressed PCR (PCR/RE) and T7 endonuclease I (T7EI) assays. The utilization of CRISPR/Cas9 to induce large chromosomal deletions employing two targets in different constructs has been reported in rice [26] and tomato [27]. However, the delivery of multiple constructs to induce genome deletions simultaneously is a challenge for crops with low transformation frequency. Obviously, highly efficient editing is more desirable, especially for those crops such as soybean that are relatively recalcitrant to *Agrobacterium*-mediated transformation.

In order to address these issues and to demonstrate simultaneous editing of homeologous genes, which is of particular relevance in tetraploid soybean, we developed a highly efficient CRISPR/Cas9 editing process employing two customized gRNAs. The utility of our approach was demonstrated by simultaneously editing both *GmFAD2–1A* and *GmFAD2–1B*. All T0 transformation events showed induced mutations in either or both *GmFAD2* genes, and notably, 50% of the T0 primary plants harbored homozygous or biallelic mutations in either or both genes. We observed various types of *GmFAD2* mutant alleles that were heritable in T1 progenies. As expected, seeds derived from double homozygous mutant plants showed a typical high oleic acid (83.3%) phenotype compared to wild-type seeds (20.2%). Lastly, ‘transgene-free’ double *GmFAD2* homozygous mutants were readily obtained at the T1 generation, which under the present USDA regulation, would be considered non-regulated yet value-added high oleic soybean lines.

## Results

### Vector construction and confirmation of target sites in soybean hairy roots

**Fig. 1:**
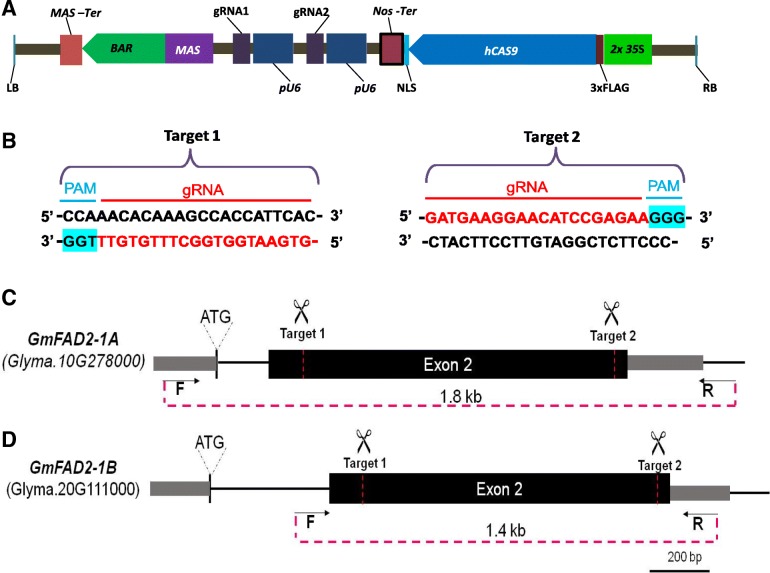
Diagram of dual gRNA CRISPR/Cas9 vector, target sequences and target locations. **a** CRISPS/Cas9 vector for soybean transformation with pFGC5941 backbone, *bar* gene as selection marker, *Cas9* sequence (h*CAS9*) and two sgRNAs. Cas9 and gRNA expression are driven by the *35S* promoter and Arabidopsis *U6* promoter (pU6). MAS, Manopine promoter. Nos, *Nopaline Synthase* terminator; MAS-Ter, *Manopine Synthase* terminator; FLAG, 3x FLAG sequences. **b** sgRNA sequences; **c** Location of dual target sites in *GmFAD2–1A*. **d** Location of dual target sites in *GmFAD2–1B*. Gene-specific primers used for PCR genotyping are indicated by arrows

Approximately 70% of soybean genes are duplicated [28] and, hence, we sought to target both *GmFAD2–1A* and *GmFAD2–1B* to demonstrate the facility by which multiple homeologues could be edited simultaneously. To determine the efficacy of CRISPR/Cas9 in simultaneously editing homeologous genes in soybean, we targeted *GmFAD2–1A* and *GmFAD2–1B* using the double gRNA strategy as previously described [26, 27]. The CRISPR vector used encodes *Cas9* driven by the *CaMV35S* promoter and two gRNAs driven by the Arabidopsis *U6* promoter (Fig. 1a, b). The two gRNAs were designed to guide Cas9 to simultaneously cleave two sites, spaced 1Kb apart, within the second exons of *GmFAD2–1A* and *GmFAD2–1B* (Fig. 1c). Transformation with this vector would be expected to generate double strand breaks at both target sites resulting in genomic deletions, which can be readily detected by the altered electrophoretic mobility shift of PCR-generated amplicons. Hence, the creation of deletions would indicate the efficacy of the two gRNAs and greatly reduces the labor and cost of genotyping. Using the online CRISPR/Cas9 guide RNA design program, CCTop [29] we identified and selected two customized gRNAs that would guide Cas9 to cleave two sites within each of *GmFAD2–1A* and *GmFAD2–1B* exons simultaneously generating deletions (approximately 1 kb) in each gene (Fig. 1c).

In order to test whether the CRISPR construct would perform properly in transgenic soybean plants, we first tested the construct by transient hairy root transformation using *Agrobacterium rhizogenesis* strain K599. The hairy root transformation system was improved from Kereszt et al., 2007 [30] so that transgenic hairy roots were formed and collected within 12 days for genotyping. Transgenic hairy roots were genotyped by PCR using gene-specific primers flanking the two target sites (Fig. 1c). Expectedly, the utilization of these primers generated PCR fragments of ~ 1.8 kb and ~ 1.4 kb for *GmFAD2–1A* and *GmFAD2–1B*, respectively, using wild-type DNA, while ~ 0.8 kb and ~ 0.4 kb PCR products were observed using DNA derived from transformed hairy roots (Additional file 1: Figure S1). We detected mobility- shifted bands in two of five bulked DNA samples when specific primers of *GmFAD2–1A* were utilized. Likewise, three of five samples showed mobility-shifted bands for *GmFAD2–1B*. Importantly, we found two samples that showed mutant bands for both *GmFAD2* genes, which when sequenced, revealed deletions of DNA sequences between targets 1 and 2 (data not shown). These results indicated that the transgene-encoded Cas9 and gRNAs were able to efficiently induce double-strand breaks at both target sites in both *GmFAD2* genes.

### High frequency of CRISPR-induced mutations in *GmFAD2* genes

We generated 24 primary (T0) transgenic events in a single independent transformation attempt (200 explants) following the *Agrobacterium tumefaciens* mediated soybean cotyledonary-node system modified from published protocols [31, 32]. Transgene integration was determined by herbicide leaf painting and PCR screening of T0 events for the presence of both *bar* and Cas9 genes. We then analyzed ten randomly selected transgenic events for the presence of altered electrophoretic mobility of PCR amplicons, indicative of genetic deletions.

**Fig. 2:**
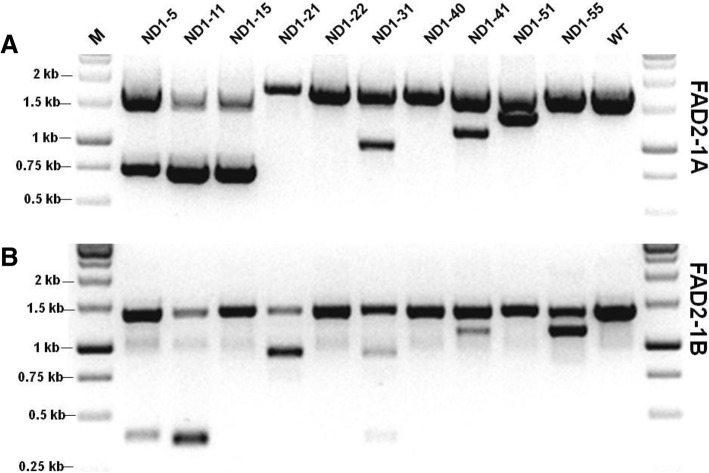
Identification of *GmFAD2* gene deletions in T0 soybean transformants using PCR band shift assay. **a** Gel electrophoresis of PCR amplicons using specific primers for *GmFAD2–1A*. **b** Gel electrophoresis of PCR amplicons using specific primers for *GmFAD2–1B*

**Fig. 3:**
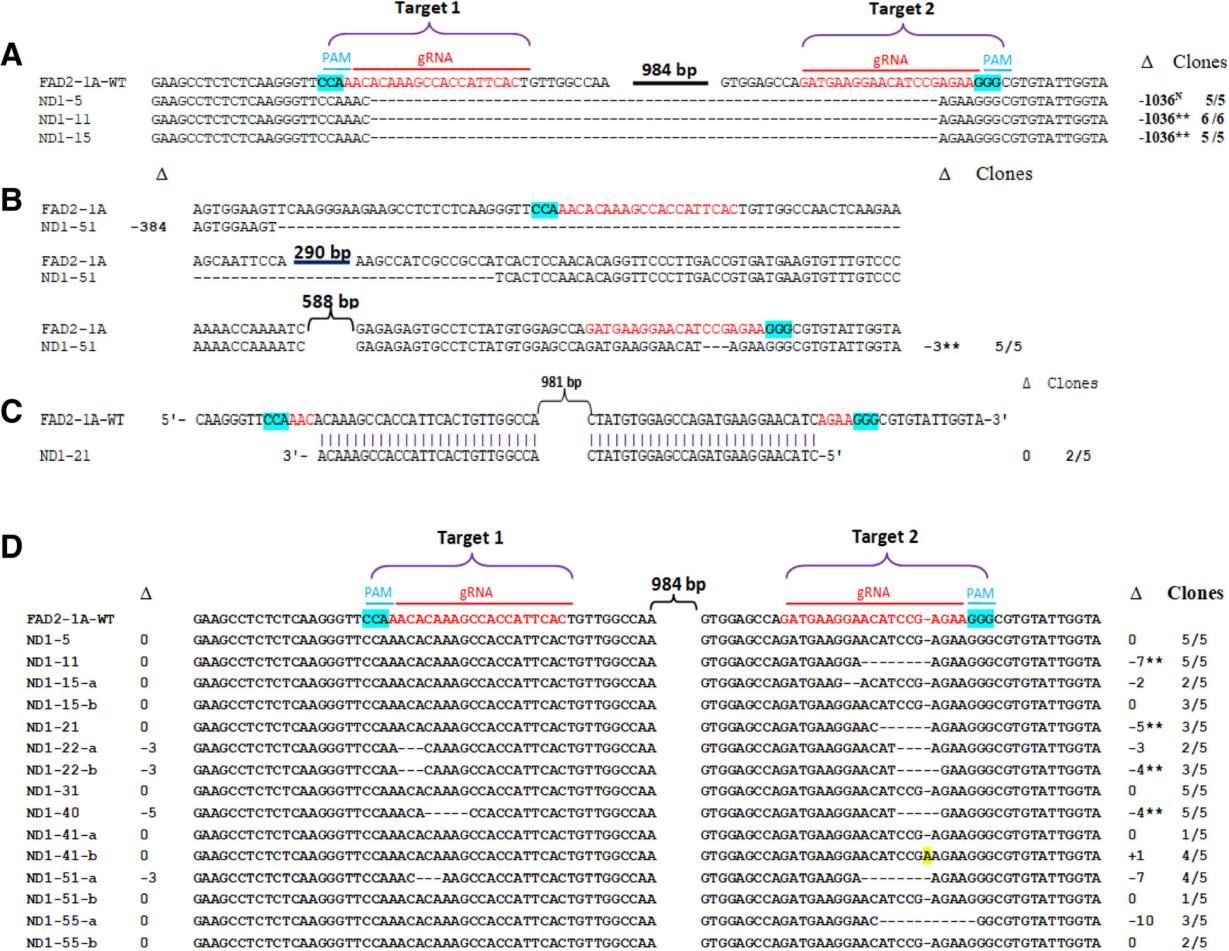
CRISPR/Cas9-induced mutations in *GmFAD2–1A* in T0 transgenic plants. **a** Expected deletions due to excision of DNA sequences between the PAM sites. **b** Partial deletions in transgenic line ND1–5. **c** Inversion of *GmFAD2–1A* sequences between the PAM sites. **d** Small deletions/insertion in target 1 and/or target 2. The number of PCR amplicons giving mutant sequences out of the total amplicons sequenced (Clones) and the indels (Δ) detected in target 1 (left values) or target 2 (right values) for each of the ten T0 events are indicated. Inherited indels in T1 and/or T2 progenies are marked by ** and chimeric indels are marked by N. -, deleted nucleotides; +, inserted nucleotides; 0, no deletion or insertion; a, b, different alleles detected in each T0 event

**Fig. 4:**
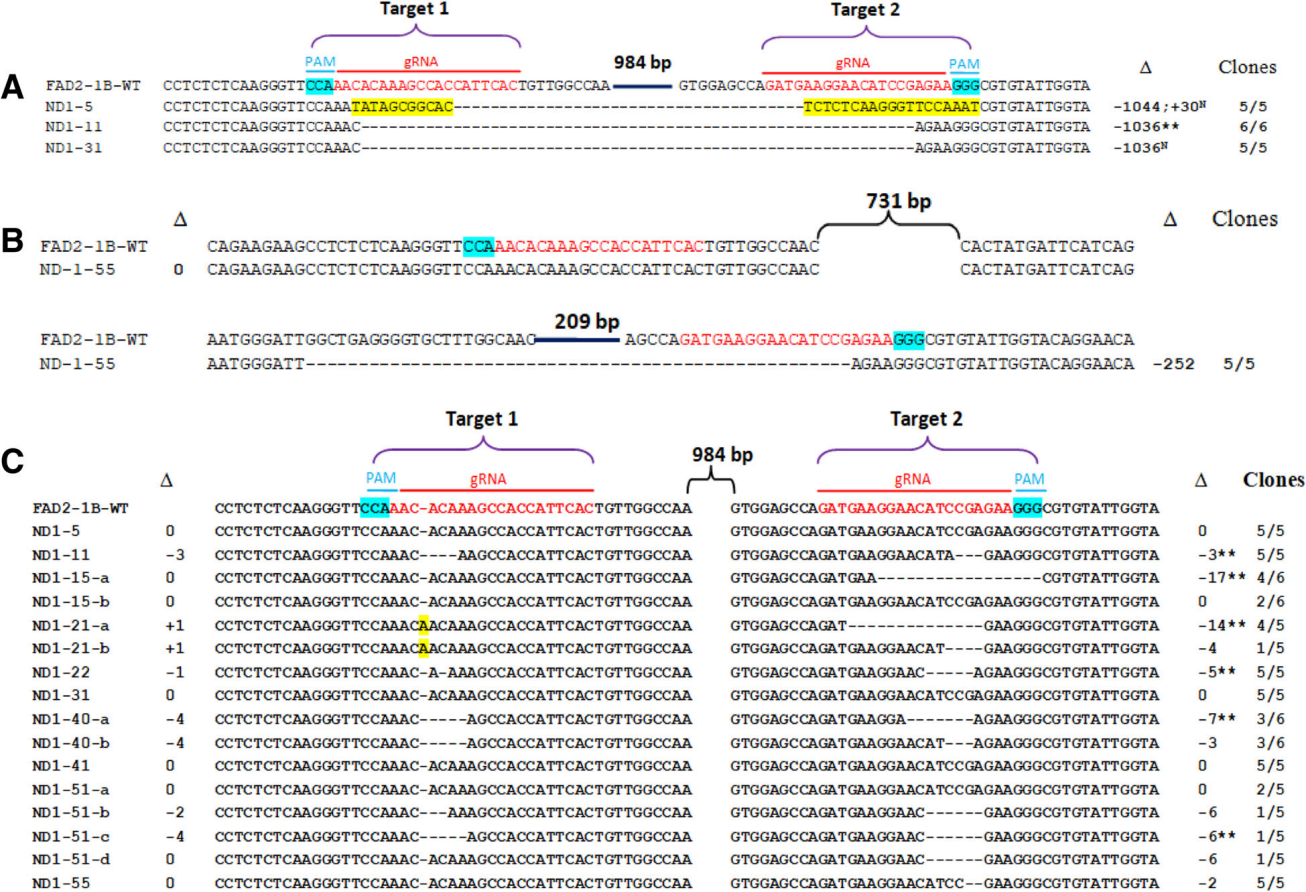
CRISPR/Cas9-induced mutations in *GmFAD2–1B* in T0 transgenic plants. **a** Expected deletions due to excision of DNA sequences between the PAM sites. **b** Partial deletions in transgenic line ND1–55. **c** Small deletions/insertion in target 1 and/or target 2. The number of PCR amplicons giving mutant sequences out of the total amplicons sequenced (Clones) and the indels (Δ) detected in target 1 (left values) or target 2 (right values) for each of the ten T0 events are indicated. Inherited Indels in T1 and/or T2 progenies are marked by ** and chimeric Indels are marked by N. -, deleted nucleotides; +, inserted nucleotides; 0, no deletion or insertion; a, b, different alleles detected in each T0 event

The expected CRISPR/Cas9-induced deletions in *GmFAD2–1A* (~ 0.8 kb amplicons) were detected in events ND1–5, ND1–11 and ND1–15 (Fig. 2a), whereas the expected deletions (0.4 kb) in *GmFAD2–1B* were detected in events ND1–5, ND1–11 and ND1–31 (Fig. 2b). Subsequent sequencing of the gel-shifted PCR amplicons showed the expected deletions (1036 bp in size) of *GmFAD2–1A* in transgenic events ND1–5, ND1–11 and ND1–15 (Fig. 3a). Similar 1036 bp deletions in *GmFAD2–1B* were confirmed in events ND1–5, ND1–11 and ND1–31 (Fig. 4a). In these deletions, the cleavage sites were located at the third nucleotide upstream of the Protospacer Adjacent Motif (PAM) sequence on target1 and at the fourth nucleotide upstream of the PAM sequence on target 2. Event ND1–5 contained a large deletion (1044 bp) of *GmFAD2–1B* and also a 30 bp inserted fragment (Fig. 4a). These results indicated that Cas9 cleaved the *GmFAD2* genes at the two target loci simultaneously. Our PCR genotyping results also showed unexpected smaller size deletions in T0 events ND1–31, ND1–41 and ND1–51 for *GmFAD2–1A* and in events ND1–21, ND1–31, ND1–41 and ND1–55 for *GmFAD2–1B* (Fig. 2). Sequencing data of the lower mobility PCR fragments again confirmed the partial deletions of *GmFAD2* genes in these events (Figs. 3b and 4b and Additional file 1: Figure S2). The partial deletions included the loss of fragments (from ~ 200 bp to > 800 bp) due to DSBs (double strand breaks) in either target 1 or target 2. The sequencing results were consistent with the observed gel mobility of the corresponding PCR fragments. Overall, seven of the ten T0 events we analyzed harbored deletions in either or both *GmFAD2* genes that were easily detected by electrophoretic mobility shift of PCR amplicons.

The generation of a variety of on-site mutations is a common occurrence using CRISPR/Cas9 [33, 34] Therefore, we also sequenced a number of *GmFAD2* PCR fragments generated from transgenic lines that showed no change in electrophoretic mobility relative to wildtype (WT). Sequencing of these PCR fragments identified a variety of small deletions at the target sites of either *GmFAD2–1A* or *GmFAD2-B* (Figs. 3d and 4c). The small deletion sizes varied from 1 to 5 nucleotides on target 1 and from 2 to 17 nucleotides on target 2. One nucleotide insertions were observed on target 1 of *GmFAD2–1B* in line ND1–21 and on target 2 of *GmFAD2–1A* in line ND1–41. Transgenic events ND1–5 and ND1–31 showed no small insertion or deletion in both target sites of *GmFAD2–1A* gene. Moreover, no small indels (insertions/deletions) were found affecting the *GmFAD2–1B* gene in transgenic events ND1–5, ND1–31 and ND1–41. Interestingly, we found inverted regions with the *GmFAD2–1A* gene in transgenic line ND1–21 (Fig. 3c). No deletion or insertion was observed in both targets. However, a large fragment (1306 bp) was inverted between the two target sequences. This result may arise from DSBs and repair occurred simultaneously at both targets of *GmFAD2–1A* gene [19, 26, 27, 35]. Our sequencing data also showed differences in the editing frequency at the two target loci (Figs. 3 and 4 and Additional file 1: Figure S2). Editing of *GmFAD2–1A* and *GmFAD2–1B* at target 1 and target 2 occurred at 43 and 57%, respectively. In addition to the expected large deletions, small indels were found at frequencies of 43.3% (26/60 sequences) in *GmFAD2–1A* at target 1 and 68.3% (41/60 sequences) at target 2. Likewise, sequencing results to identify mutations in *GmFAD2–1B* gene indicated that the indels were induced at a frequency of 45.2% (28/62) at target 1 and 66.1% (41/62) at target 2.

In summary, genotyping of T0 events, either by PCR gel electrophoresis and by DNA sequencing, indicated that 100% of tested transformation events carried mutations in either or both *GmFAD2* genes (Figs. 3 and 4, Table 1, Additional file 1: Figure S2). Of the ten T0 events, four (or 40%) showed null mutations in both *GmFAD2–1A* and *GmFAD2–1B* genes, namely, events ND1–11, ND1–21, ND1–22 and ND1–40 (Table 1). These results reflect a remarkably high editing efficiency achieved through delivery of two customized gRNAs carried by a single construct.

**Table 1:** Summary of T0 genotyping data for *GmFAD2–1A* and *GmFAD2–1B*

Events	T0 generation			
	Bar	Cas9	*GmFAD2–1A* mutants	*GmFAD2–1B* mutants
ND1–5	+	+	Heterozygous^a^	Heterozygous^a^
ND1–11	+	+	Biallelic	Biallelic
ND1–15	+	+	Chimeric^b^	Heterozygous
ND1–21	+	+	Biallelic	Biallelic^c^
ND1–22	+	+	Biallelic	Homozygous
ND1–31	+	+	Heterozygous^a^	Heterozygous^a^
ND1–40	+	+	Homozygous	Biallelic
ND1–41	+	+	Chimeric^b^	Heterozygous
ND1–51	+	+	Chimeric^b^	Chimeric^b^
ND1–55	+	+	Heterozygous^d^	Biallelic^d^

^a^Mutant allele(s) was not transferred to progenies

^b^At least one mutant allele was transferred to progenies

^c^Two mutant alleles were stably transferred to progenies; the 3rd allele (−4 in Fig. 4c) was not inherited and likely an artifact of sequencing

^d^Inheritance of mutant alleles were not determined

### Development of genetic markers and inheritance of *GmFAD2* mutant alleles

Genetic markers that identify mutant alleles are critical for effective marker assisted breeding studies. The potential markers should save time, labor and accelerate genotyping procedure in progeny. Therefore, we developed different genetic markers to identify the various *GmFAD2* mutant alleles in subsequent generations. We used specific *GmFAD2* flanking primers as PCR-based markers for deletions easily observed by gel electrophoresis mobility shift. Using this method, we confirmed the inheritance of the large deletions in *GmFAD2–1A* or *GmFAD2–1B* in T1 progenies of events ND1–11, ND1–15, ND1–21, ND1–41 and ND1–51. We also confirmed the inheritance of large *GmFAD2* deletions in T2 progenies of event ND1–11 by PCR and sequencing (Additional file 1: Figures S3 and S4).

**Fig. 5:**
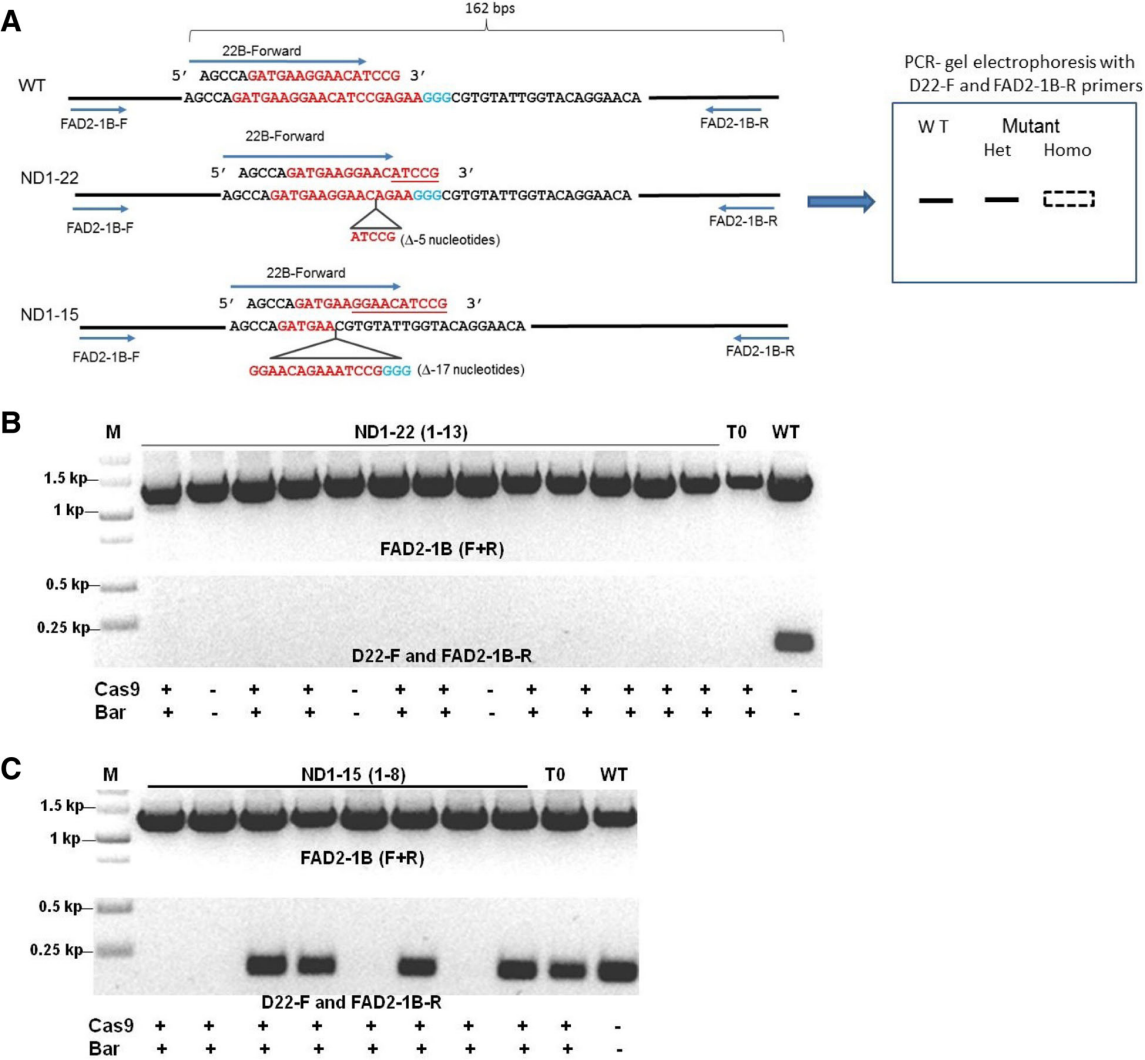
Inheritance and segregation of *GmFAD2* small deletions at T1 generation. **a** Diagram of PCR-based genotyping for small deletions. An example of Indel-specific primer, D22-F, designed for transgenic events ND1–22 and ND1–15 is shown. **b** PCR-based genotyping of T1 plants from transgenic event ND1–22 showing homozygous mutation in *GmFAD2–1B*. **c** PCR-based genotyping of T1 plants from transgenic event ND1–15 showing heterozygous mutation in *GmFAD2–1B*. PCR-based genotyping results for *bar* and *Cas9* genes are shown in (**b**) and (**c**). (+) gene was detected, (−) gene was not detected

To facilitate the genotyping of small indels, we performed PCR amplifications using *GmFAD2*-specific and indel-specific primer pairs (diagrammed in Fig. 5a). The indel-specific primers were designed to anneal only to wild-type sequences, producing amplicons on wild-type but not on mutant genomic DNA templates. Since most of the small deletions we detected were overlapping (Figs. 3d and 4c), an indel-specific primer pair can be used to genotype the progenies of multiple T0 events. For example, using the primer pair D22-F and GmFAD2–1B-R, we confirmed the heritability of the target 2 small deletions in events ND1–22 (Fig. 5b) and ND1–15 (Fig. 5c). These results are consistent with our sequencing data that the ND1–22 and ND1–15 T0 events are homozygous and heterozygous mutants, respectively, for *GmFAD2–1B* (Fig. 4a, b). Using various primer pairs (Additional file 1: Table S1), we were also able to confirm the inheritance of small deletions (Fig. 3d) in *GmFAD2–1A* for events ND1–11, ND1–21, ND1–22 and ND1–40 (Data not shown). Likewise, small deletions in *GmFAD2–1B* were passed to T1 progenies of transgenic events ND1–15, ND1–21, ND1–22, ND1–40 and ND1–51 (Fig. 4c).

Overall, the employment of these genetic markers indicated that 77.8% (7/9) tested T0 events (ND1–11, ND1–15, ND1–21, ND1–22, ND1–40, ND1–41 and ND1–51) transmitted the *GmFAD2* mutations to the T1 generation. Moreover, we tested a total of 23 *GmFAD2* mutant alleles, of which 20 (87%) were inherited in T1 and/or T2 progenies (Figs. 3 and 4 and Additional file 1: Figures S2 and S4). Genotyping of T1 and/or T2 progenies confirmed that four of the ten T0 events were indeed homozygous or biallelic mutants for both *GmFAD2* genes, whereas two events (ND1–5 and ND1–31) showed non-heritable mutations for both genes (Table 1).

### Fatty acid profile of double homozygous *GmFAD2–1A* and *GmFAD2–1B* mutant seeds

**Fig. 6:**
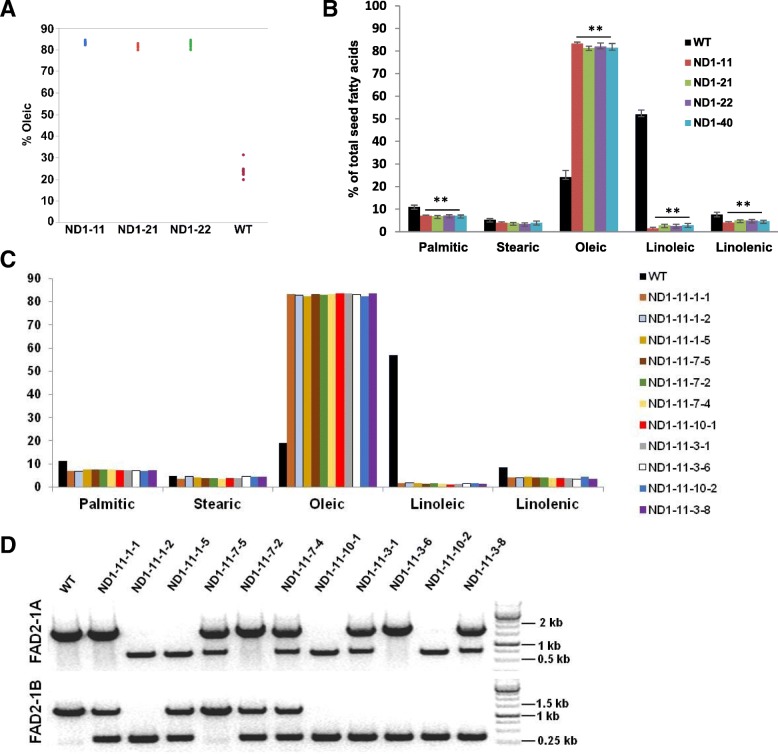
Fatty acid profiles of seeds derived from T0 events carrying double homozygous or biallelic *GmFAD2* mutations. **a** Oleic acid phenotype of individual T1 seeds from T0 events and WT Maverick. *n* = 9 or 10 seeds. **b** Mean fatty acid content of T1 seeds. *n* = 9 or 10 seeds; error bars, standard deviations. **c** Fatty acid content of individual T2 seeds derived from event ND1–11 carrying biallelic mutations in both *GmFAD2* genes. **d** PCR-based genotyping of individual ND1–11 T2 seeds shown in (**c**) for *GmFAD2–1A* and *GmFAD2–1B* mutations

Our genotyping data showed that four of ten T0 events carried CRISPR-induced homozygous mutations (either monoallelic or biallelic at T1 generation) in both *GmFAD2–1A* and *GmFAD2–1B*. To confirm the observed high efficiency of targeted mutagenesis, we performed fatty acid profile analysis of individual T1 seeds derived from the double *GmFAD2* homozygous lines ND1–11, ND1–21, ND1–22 and ND1–40 wild type Maverick seeds. Seeds derived from these lines showed the expected high and low levels of oleic acid and linoleic acid, respectively, compared to wild type Maverick seeds (Fig. 6a, b). On average, oleic acid content increased dramatically from ~ 20% in wild type seeds to over 80% in all double-null mutant lines tested. Concomitantly, linoleic acid levels dropped to 1.3–1.7% compared to 55.3% of wild-type plants. Palmitic and linolenic acid levels were also significantly reduced in the double *GmFAD2* mutants, whereas stearic acid levels were comparable to wildtype (Fig. 6b).

We also analyzed the fatty acid profile of T2 seeds derived from event ND1–11 carrying biallelic mutations in both *GmFAD2–1A* (∆-1036 and ∆-7, Fig. 3) and *GmFAD2–1B* (∆-1036 and ∆-3/∆-3, Fig. 4). This line was selected to further confirm our genotyping results and to determine if the in-frame ∆-3/∆-3 *GmFAD2–1B* allele encodes a functional protein. Randomly selected T2 seeds were analyzed from four T1 plants displaying different combinations of *GmFAD2* alleles: ND1–11-10 (∆-1036 for both *GmFAD2* genes), ND1–11-7 (∆-7*GmFAD2–1A*, ∆-1036 *GmFAD2–1B*), ND1–11-1(∆-1036 and ∆-7 *GmFAD2–1A*; ∆-1036 and ∆-3/∆-3 *GmFAD2- 1B*) and ND1–11-14 (∆-1036 and ∆-7 *GmFAD2–1A*; ∆-1036 *GmFAD2–1B*). Each T2 seed was chipped for fatty acid analysis and then planted for PCR-based genotyping. The fatty acid analysis data showed that the T2 seeds, regardless of encoded *GmFAD2* allele, had nearly identical profiles that were consistent with the detected double biallelic mutations in ND1–11 (Fig. 6c, d). Moreover, the high oleic content in ND1–11–7-5, which is homozygous for the ∆-3/∆-3 *GmFAD2–1B* allele, indicated that this in-frame deletion allele causes a null phenotype. To determine if the increased oleic acid content in the double *GmFAD2* mutants resulted in altered accumulation of fatty acids (total oil) and protein in seeds, we also evaluated these traits in T2 and T3 progenies derived from ND1–11. We found no significant differences in the seed protein or oil content between ND1–11and Williams 82 or Maverick wild-type cultivars (Additional file 1: Table S4).

### Transgene-free high oleic CRISPR mutants

One major advantage of employing CRISPR/Cas editing in crop improvement is the ability to segregate-out transgenic sequences from the induced genetic lesions. To identify homozygous *GmFAD2* mutant lines without transgenes, we screened T1 plants derived from the high oleic acid event ND1–22 for the absence of *bar* and *Cas9* genes. We found that three out of 13 T1 plants derived from ND1–22 showed the absence of both *bar* and Cas9 genes (Fig. 5b). In addition, seeds of four T1 plants from another high oleic acid event, ND1–11, were planted and screened for the absence of *bar* and *Cas9* by PCR and leaf-painting with 100 mg/ml glufosinate. We found that all ND1–11-14 T2 plants carried no *bar* and *Cas9* whereas ND1–11-2 and ND1–11-10 T2 progenies segregated for *bar* and *Cas9* genes (Additional file 1: Table S2). These results, therefore, demonstrated that transgene-free homozygous *GmFAD2* mutant plants were readily obtained as early as the T1 generation.

### Off-target mutations were not detected in high oleic CRISPR mutants

Two potential off-target sites in the soybean Williams 82 cultivar were bioinformatically identified using the web tool CRISPR-P (http://cbi.hzau.edu.cn/crispr/). These off-target sites contained identical PAM sequences and mismatches of 3–4 base pairs compared to the targeted sites in *GmFAD2–1A* and *GmFAD2–1B* genes (Additional file 1: Table S3). To determine if off-target mutations were induced at these sites, flanking regions were PCR-amplified from T2 plants derived from the high oleic event ND1–11. PCR amplicons from 30 T2 plants were sequenced and no mutations were detected in either off-target gene (Additional file 1: Table S3 and Figure S5).

## Discussion

CRISPR/Cas9 technology has great promise for rapid and efficient genome modification for crop improvement. It has been successfully applied for genome editing using various soybean culture systems [17–22, 24, 25]. In addition, a few reports have shown successful gene editing after stable transformation of soybean [19, 23–25]. These reports used a single guide RNA to induce mutations in either single or multiple genes. However, both mutation and mutation inheritance rates were found to be low in stable transgenic soybean plants. By contrast, high genome editing frequencies were achieved previously in other plant species when two or multiple gRNAs were deployed to induce cleavages of two adjacent target sites [26, 27, 36, 37]. Such an approach is especially attractive in editing the large, highly duplicated soybean genome where over 70% of genes are duplicated [28]. In this study, using dual gRNA to target two adjacent sites in both *GmFAD2–1A* and *GmFAD2–1B,* we performed detailed evaluation of the efficiency of inducing heritable CRISPR/Cas9 mutations in soybean. We found an extremely high editing frequency in T0 generation transgenic soybean plants*,* i.e., four of the ten T0 (40%) events we analyzed carried null mutations in both *GmFAD2–1A* and *GmFAD2–1B* (Table 1). Fatty acid profile analysis of T1 seeds derived from these events showed high oleic acid (~ 80%) and low linoleic acid content, confirming that these events indeed carried double homozygous mutations in the *GmFAD2* genes (Fig. 6a, b). This is in sharp contrast to a single gRNA-mediated gene editing previously reported in soybean where homozygous mutants were obtained only in the T1 or T2 generations [19, 23, 24]. Furthermore, the induced *GmFAD2* mutations were transmitted to subsequent generations at high frequencies, i.e., 87% (20/23) of the mutant alleles tested were inherited in progenies (Figs. 3 and 4 and Additional file 1: Figure S2) and 77.8% (7/9) of T0 events showed heritable mutations in either or both *GmFAD2* genes (Table 1). Previous efforts using CRISPR/Cas9 system resulted in approximately 50% (two of four T0 events) of the mutations showing clear inheritance in subsequent generations [24], whereas gene editing using the TALEN system led to 50% transmission of both *GmFAD2–1A* and *GmFAD2–1B* mutations to T1 progenies [13]. The production of chimeric mutations was also reported as a problem that reduced the heritable transmission of mutant alleles in previous CRISPR/Cas9 studies in soybean [21, 24, 25]. In contrast, we were able to induce high frequency of heritable T0 mutations, which then enabled us create transgene-free high oleic soybean genotypes with multiple *GmFAD2–1A* and *GmFAD2–1B* alleles at the T1 generation.

The efficiency of inducing mutations using CRISPR/Cas9 is largely dependent on the induction of DSBs at the selected target sites. Therefore, prior to stable transformation, we confirmed the ability of our CRISPR/Cas9 construct to simultaneously cut at the dual target sites using hairy root transformation (Additional file 1: Figure S1). This confirmatory step, which took around 2 weeks and required no additional cloning steps, is necessary to ensure high frequency of editing in stably-transformed soybean plants. Moreover, utilization of two customized gRNAs created defined deletions from simultaneous cleavage at the two adjacent target sites, as well as on-site indels at either or both targets (Figs. 3 and 4 and Additional file 1: Figure S2). We also found one T0 event carrying an inverted genomic region between the two target sites of *GmFAD2* genes, presumably from the simultaneous cleavage at both target sites followed by the non-homologous end joining (NHEJ) repair. Similar to the previous report in rice [35], most T0 events carried biallelic or heterozygous mutations. The observed large partial deletions (ranging 200 bp to > 800 bp) at target 1 and target 2 site were not observed in previous studies utilizing dual gRNA CRISPR/Cas9 system [27, 35, 38–40]. The distance between the two editing sites influences not only the size of deletions generated but also editing efficiency. For example, a high frequency of large deletions was obtained in rice when the two adjacent targets were close (50–200 bp) [26, 35], whereas the frequency of this type of mutation was low as the distance between the two target sites increased [24, 38]. In our study, the distance between the two target sites of *GmFAD2* genes was ~ 1 kb resulting in four of ten T0 events displaying large deletions. Overall, our sequencing results showed that editing of *GmFAD2–1A* and *GmFAD2–1B* at target 1 and target 2 occurred at 43 and 57%, respectively. Both target sequences contain 45% G and C nucleotides (Fig. 1b) and hence, the higher editing frequency at target 2 is not due to differences in GC content but rather to differences in target site and/or PAM sequences.

Increasing the monounsaturated fatty acid component in seeds improves stability of soybean oil quality and processed food. The GmFAD2 enzyme catalyzes the conversion of oleic acid to linoleic acid. Therefore, the loss of its function leads to reduction of both linoleic and α-linolenic acids while increasing accumulation of oleic acids. In previous reports, either homozygosity for loss of function mutations or silencing both *GmFAD2–1A* and *GmFAD2–1B* genes resulted in an increase in oleic acid levels of soybean seeds [7, 8, 10–13, 41]. Therefore, it is not surprising that the same phenotype was found when the *GmFAD2–1A* and *GmFAD2–1B* genes were mutated using CRISPR/Cas9. However, this study clearly demonstrated the speed and efficiency by which gene editing was applied to generate non-transgenic soybean genotypes with an improved seed trait.

## Conclusions

Using the *GmFAD2- 1A* and *GmFAD-1B* genes are targets, we demonstrated that dual gRNA CRISPR/Cas9 system induced high frequency of heritable mutations in homeologous soybean genes. The use of two gRNAs to target each gene induced mutations at both or either target sites, thus enabling us to achieve the high frequency of double homozygous mutations in *GMFAD2–1A* and *GmFAD2–1B* at the T0 generation. The two adjacent target sites also created large deletions that can be genotyped by PCR, allowing a fast method of confirming the efficacy of the gRNAs and the inheritance of the mutations in subsequent generations. Soybean is allotetraploid and is recalcitrant to transformation and thus, transgenic plants are costly and take a long time to produce. Therefore, this study provides encouraging data to soybean researchers on the utility of targeted genome editing, e.g., CRISPR/Cas9, as a cost-effective approach for generating genetic modifications suitable for downstream soybean breeding efforts, field propagation and eventual germplasm/cultivar release.

## Methods

### CRISPR/Cas9 vector construction with two customized gRNAs

The human codon-optimized Cas9 gene (35S-Cas9-SK), driven by the CaMV 35S promoter and the chimeric single guide RNA (AtU6–26-SK), driven by the AtU6–26 promoter were gifts from Jian-Kang Zhu [42]. The *bar* gene, driven by mannopine synthase promoter of the binary vector pFGC5941, was used as selection marker for soybean stable transformation. Soybean specific single-guide RNA sequences were designed using the web tools: CCTop [29]. Two gRNAs (gRNA1 and gRNA2) were used to create defined deletions ~ 1049 bp within the exon of each gene (*GmFAD2–1A* and *GmFAD2–1B*). For each gRNA, a pair of DNA oligonucleotides (Additional file 1: Table S1) was synthesized by Integrated DNA Technologies, Inc. (IDT; Coralville, IA) and annealed to generate dimers. Subsequently, the annealed DNA was cloned into *BbsI* sites of pAtU6–26-SK to create pSK-AtU6–26-gRNA, and sequence integrity was confirmed by Sanger sequencing. To obtain functional Cas9 expression construct for targeted mutagenesis, pSK-AtU6–26- gRNA1 was cut with *BamHI-SpeI*, pSK-AtU6–26- gRNA2 was cut with *BamHI- EcoRI*, and 35S-Cas9-SK was digested with *HindIII-SpeI*. These 3 fragments were assembled into pFGC5941 by *HindIII-EcoRI* restriction digestion followed by ligation to give the pFGC-GmFAD2-CRISPR construct. The positive plasmids were introduced into *Agrobacterium tumefacien* strain AGL1 by electroporation and used for subsequent soybean stable transformations.

### Soybean hairy root transformation

Soybean (*Glycine max*) seeds of cultivar ‘Williams 82’ [43] was used for hairy root transformation as described by Kereszt et al., 2007 [30] with some modifications. Briefly, seeds were surface-sterilized with 70% ethanol for 1 min, followed by 10% chlorox for 10 min. Seeds were then rinsed 6 times with sterile deionized water and germinated in 3″ pots (with 3 seeds per pot) filled with 1:1 (v/v) mixture of sterilized perlite and vermiculite (Hummert International, St Louis, MO). Pots were watered regularly with nitrogen-free plant nutrient solution B&D [44] and maintained in a controlled environment plant growth chamber (16 h light; 27 °C; 80% humidity). Three and a half day old soybean seedlings were infected by *Agrobacterium rhizogenes* K599 harboring pFGC-GmFAD2-CRISPR construct. Twelve days after infection, hairy roots produced from the infected sites were collected and genotyped by PCR using primers flanking the target sites in *GmFAD2–1A* and *GmFAD2–1B* as described below. Genotyping was done on hairy roots derived from five infected plants.

### Stable soybean transformation and transgene confirmation

Elite soybean genotype “Maverick” [45] was transformed using *Agrobacterium-*mediated cotyledon node system. The transformation procedure was modified from previous protocols [31, 32]. Putative transgenic soybean plants were screened by herbicide leaf-painting of fully expanded leaves at three vegetative stages (V3, V4 and V5) by swiping 100 mgl^− 1^ glufosinate-ammonium solution onto the upper leaf surface. Genomic DNA was extracted from leaves of herbicide resistant plants using CTAB method [46] to confirm the presence of *bar* and *Cas9* genes by PCR using gene specific primers (Additional file 1: Table S1). PCR amplifications were done once for each DNA sample.

### Plant materials, plant growth conditions and seed storage

Williams 82 and Maverick soybean seeds used for hairy root and stable transformations, respectively, were obtained from the Missouri Soybean Foundation Seed, Columbia, Missouri, U.S.A. Transgenic and wild-type control plants were grown in 2017–2018 in glass houses at the University of Missouri, Columbia, MO, with a photoperiod of 18 h light/ 6 h dark, and day/light temperature of 26/22 °C. Each plant was grown in a three-gallon pot and fertilized with Peters 20–20-20 fertilizer (Hummert International, cat. no. 07–5400-1) following the manufacturer’s specifications. Pots containing transgenic and control plants were arranged following a Complete Random Design. For seeds derived from field growth conditions, plants were grown at the University of Missouri Bradford Research Center, Columbia, Missouri. Mature seeds were harvested, and stored in a long-term seed storage room (4 °C and 40% humidity) in the Ernie and Lottie Sears Plant Growth Facility, University of Missouri, Columbia.

### Identification of induced mutations using PCR and sequencing analyses

The regions spanning two targets of *GmFAD2* genes were amplified using Phusion® High-Fidelity DNA Polymerase (NEB, Ipswich, MA, USA) with different primer pairs for *GmFAD2–1A* or *GmFAD2–1B* (Additional file 1: Table S1). The PCR products were separated by 1% agarose gel and, purified from gel and ligated to pGEM®-T Easy Vector Systems (Promega, Madison, WI, USA) for sequencing. The sequencing was performed utilizing a 3730xl 96-capillary DNA Analyzer with Applied Biosystems Big Dye Terminator cycle sequencing chemistry (ThermoFisher, Waltham, MA, US) at University of Missouri DNA Core. The sequences of transgenic and wild-type plants were aligned using online program MUSCLE (https://www.ebi.ac.uk/Tools/msa/muscle/) to characterize the mutations induced by CRISPR/Cas9. The same sequencing approach was utilized to identify the inheritance of the mutations at T1 and T2 generations. In addition, Indel-specific primers were designed based on sequencing results and used to confirm the transmission of small deletions in progenies (Additional file 1: Table S1).

### Determination of seed protein and oil content

Approximately ten grams of soybean seeds were ground using LM 3310 seed grinder (Perten Instruments®, Sweden). Protein and oil content in the ground samples were measured using a DA 7250 Near Infrared Analyzer spectrometer (NIRS) (Perten Instruments®, Sweden) at the Bay Farm Research Facility, Columbia, MO.

### Fatty acid analysis using gas chromatography

Transgenic soybean *GmFAD2* mutant and wild-type (cv. Maverick) plants were grown until plant maturity in a greenhouse in Life Science Center, University of Missouri – Columbia, MO. Data for individual fatty acid contents (palmitic, C16:0; stearic, C18:0; oleic, C18:1; linoleic, C18:2; linolenic, C18:3) were obtained using 10–30 individual mature seeds from each event. Seed samples were manually crushed, and total oils were extracted using 1 mL of chloroform-hexane-methanol at ratio of 8:5:2 (v/v/v) overnight. A subset of 150 μL fatty acid was extracted with oil/chloroform-hexane-methanol by adding 75 μL methylating reagent [0.25 M methanolic sodium methoxide-petroleum ether-ethyl at a ratio of 1:5:2 (v/v/v)], which was then diluted with hexane to 1 ml. Capillary gas chromatograph (GC) was performed using an Agilent series 6890 instrument (Palo Alto, CA, USA), an AT-Silar capillary column (Alltech Associates, Deerfield, IL, USA) and a flame ionization detector (275 °C). A standard mixture of fatty acids (Animal and Vegetable Oil Reference Mixture 6, AOACS, Matreya, LLC, State College, PA, USA) was used for reference standards, with final values expressed as percentage of each individual fatty acid of the total seed oil.

### Statistical analysis

Seed fatty acid, protein and oil content data were analyzed using Turkey’s least significant difference in one-way ANOVA-Test using SPSS software (ver.20, Chicago, IL, USA).

## Additional file

Additional file 1: **Figure S1.** Identification of edited *GmFAD2* genes of soybean hairy roots using PCR-based genotyping. **Figure S2.** Partial deletions and insertions detected in *GmFAD2* genes. **Figure S3.** Genotyping of homozygous expected deletions and transgenes in T2 generation of event ND1–11. **Figure S4.** Inheritance of *GmFAD2* mutations in T2 progenies of event ND1–11. **Figure S5.** Sequencing results of off-target and flanking regions. **Table S1.** Primer sequences for genotyping *GmFAD2* genes. **Table S2.** Segregation of *bar* and *Cas9* in T2 progenies derived from event ND1–11. *Cas9* was detected by PCR while *Bar* was detected by PCR and leaf painting. **Table S3.** Potential off-target mutations in transgenic T2 plants derived from event ND1–11. **Table S4.** Protein and oil content in ND1-11-14 and wild type (Williams 82 and Maverick) seeds. Measurements were performed over two years under greenhouse (2017) and field (2018) conditions. (PDF 862 kb)

## Data Availability

The datasets supporting the conclusions of this article are included within the article and its additional files. The materials developed in this study are available from the corresponding author on reasonable request.
