# Supplementary material for: Demonstration of highly efficient dual gRNA CRISPR/Cas9 editing of the homeologous GmFAD2–1A and GmFAD2–1B genes to yield a high oleic, low linoleic and α-linolenic acid phenotype in soybean
Source: BMC Plant Biol. 2019 Jul 15;19:311. doi: 10.1186/s12870-019-1906-8 (PMC6632005; doi:10.1186/s12870-019-1906-8)
Supplement: Supplementary file 1 — Figure S1. Identification of edited GmFAD2 genes of soybean hairy roots using PCR-based genotyping. Figure S2. Partial deletions and insertions detected in GmFAD2 genes. Figure S3. Genotyping of homozygous expected deletions and transgenes in T2 generation of event ND1–11. Figure S4. Inheritance of GmFAD2 mutations in T2 progenies of event ND1–11. Figure S5. Sequencing results of off-target and flanking regions. Table S1. Primer sequences for genotyping GmFAD2 genes. Table S2. Segregation of bar and Cas9 in T2 progenies derived from event ND1–11. Cas9 was detected by PCR while Bar was detected by PCR and leaf painting. Table S3. Potential off-target mutations in transgenic T2 plants derived from event ND1–11. Table S4. Protein and oil content in ND1-11-14 and wild type (Williams 82 and Maverick) seeds. Measurements were performed over two years under greenhouse (2017) and field (2018) conditions. (PDF 862 kb) [file 12870_2019_1906_MOESM1_ESM.pdf]

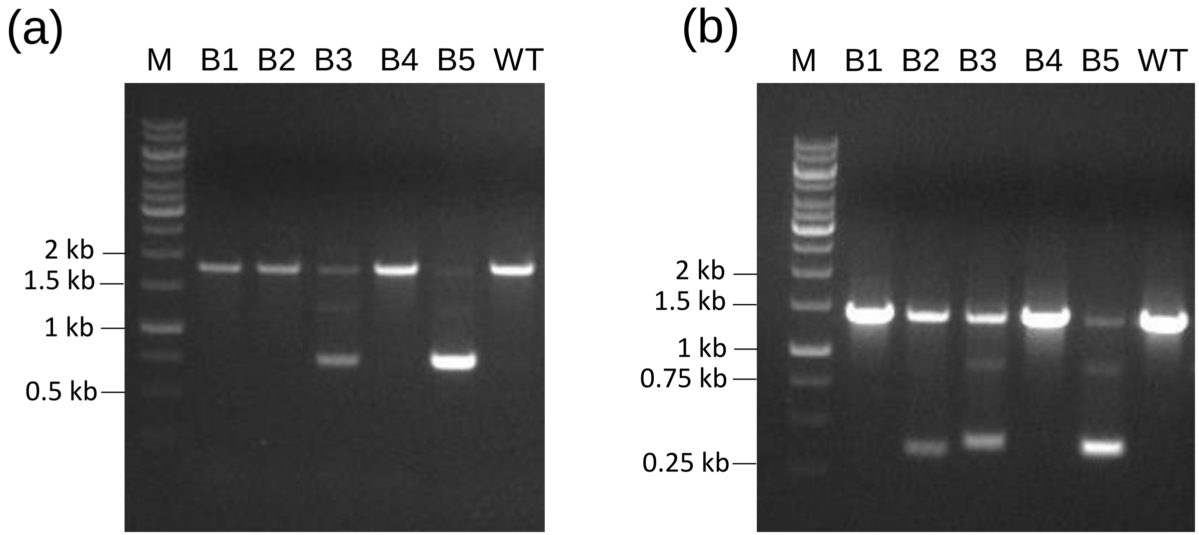


**Figure S1**. Identification of CRISPR-edited *GmFAD2* gene deletions in soybean hairy roots using PCR-based genotyping. a PCR-gel electrophoresis using specific primers for *GmFAD2-1A*. b PCR-gel electrophoresis using specific primers for *GmFAD2-1B*. Lanes B1-B5, DNA samples from pooled hairy roots of infected Plants; WT, DNA sample from uninfected roots of Williams 82; M, molecular weight markers.

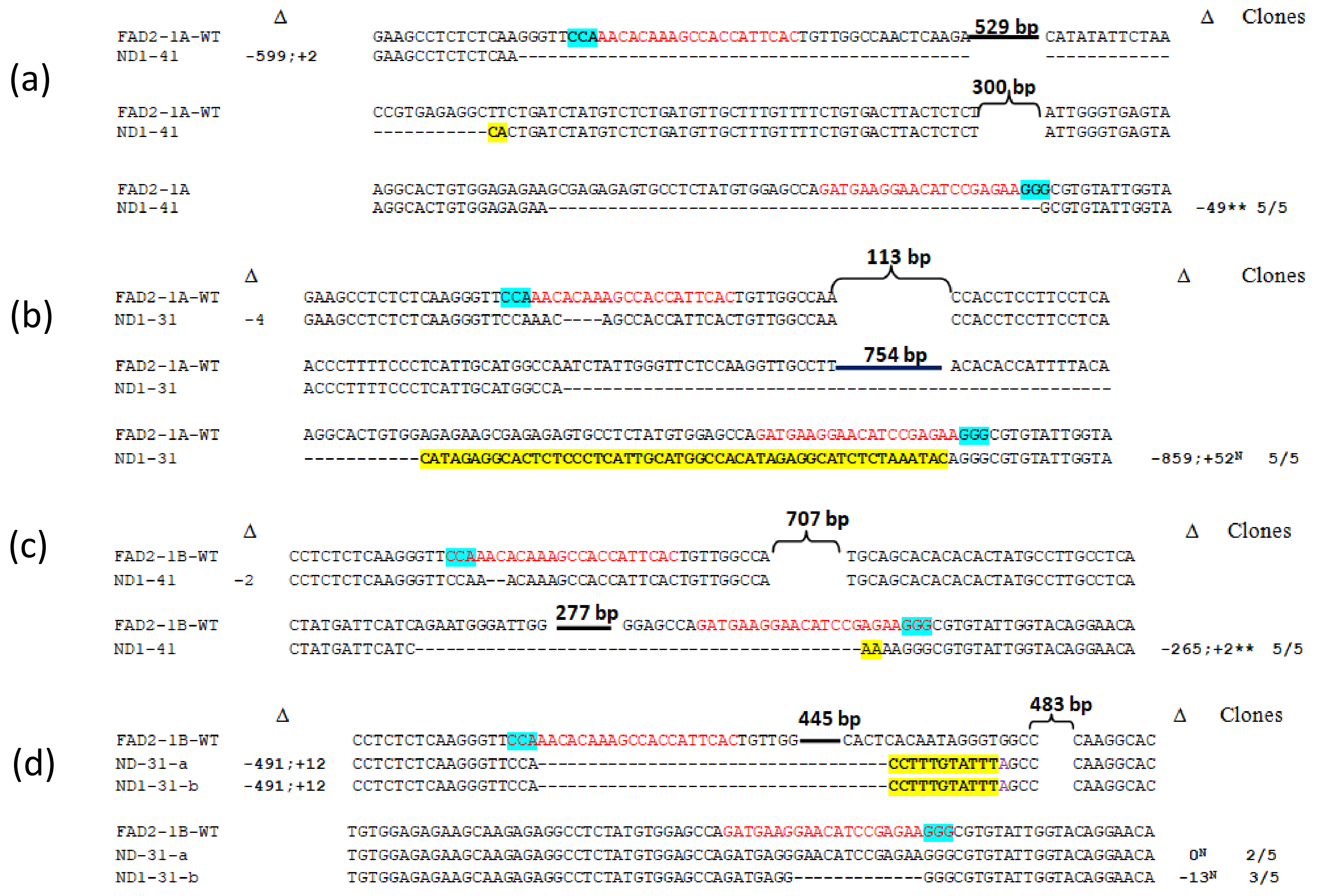


**Figure S2.** Partial deletions and insertions detected in *GmFAD2* genes. a,c Deletions in *GmFAD2-1A* and *GmFAD2-1B* in T0 event ND1-41, respectively. b,d Deletions in *GmFAD2-1A* and *GmFAD2-1B* in T0 event ND1-31, respectively. (Δ) indels in target gene sequences (negative: deleted nucleotides; positive: inserted nucleotides; 0: no deletion or insertion); (Clones) number of mutated amplicons out of total sequenced clones; small letters (a, b…) different alleles detected in each event ; ** the inheritance was confirmed in T1 and/or T2 progenies; N, non-inherited alleles.

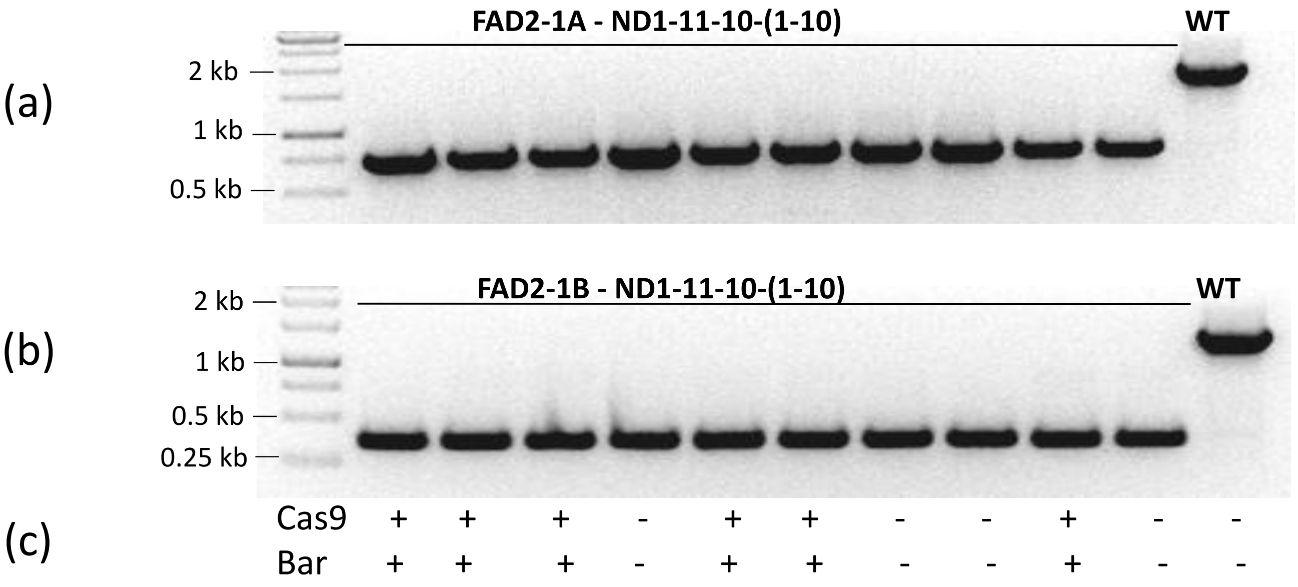


**Figure S3** . Genotyping of homozygous expected deletions and transgenes in T2 generation of event ND1-11. a PCR-based genotyping of *GmFAD2-1A.* b PCR-based genotyping of *GmFAD2-1b*. c Genotyping results for bar and Cas9 genes; (+) positive; (-) negative.

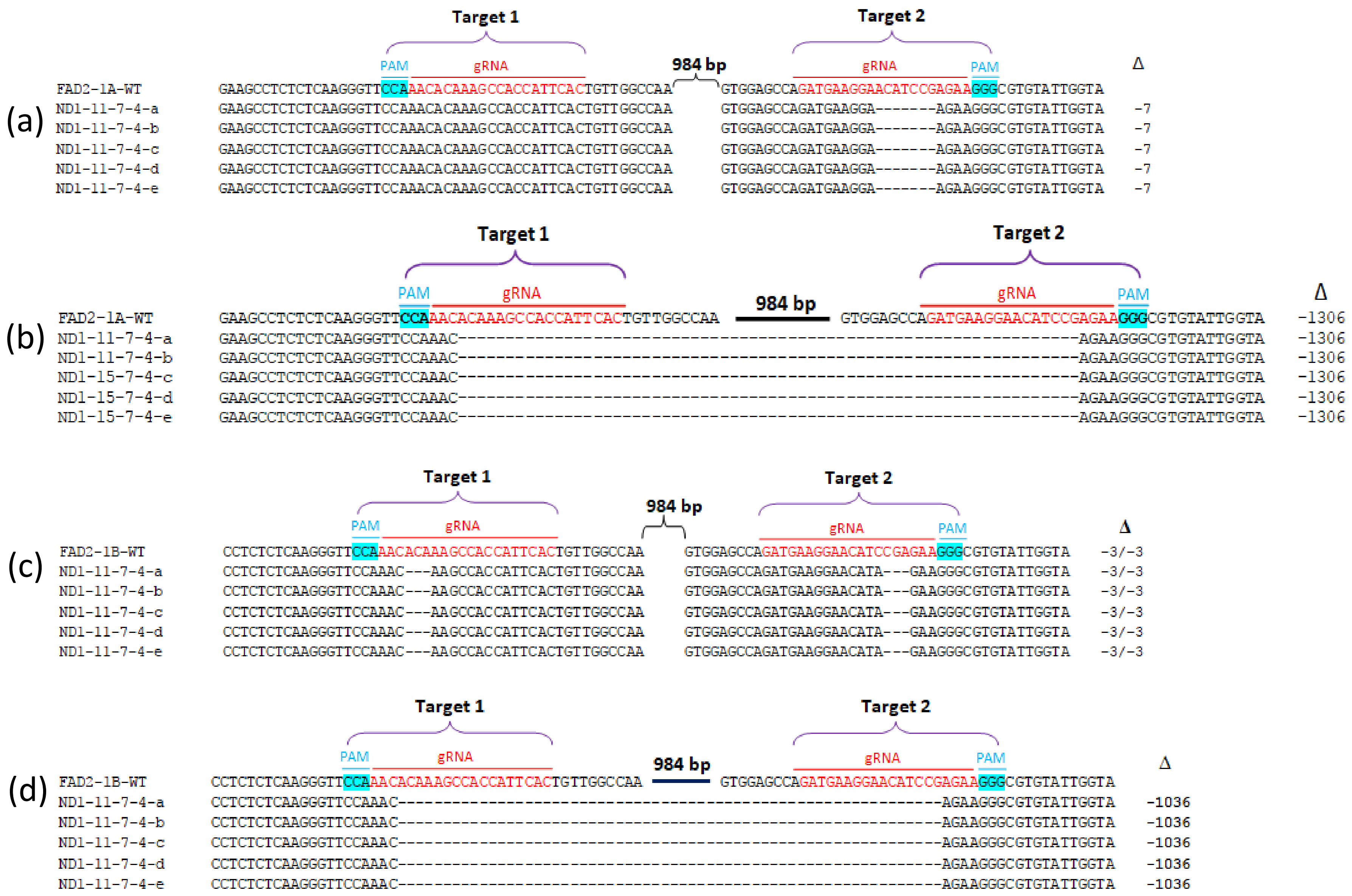


**Figure S4.** Inheritance of *GmFAD2* mutations in T2 progenies of event ND1-11. a,b Small deletions in *GmFAD2-1A* and *GmFAD2-1B*, respectively. c,d Expected deletions in GmFAD2-1A and GmFAD2-1B. Δ, Indels in target gene sequences; -, deleted nucleotides; numerator, Indels in target1; denominator, Indels in target2; small letters (a,b…) sequenced clones.

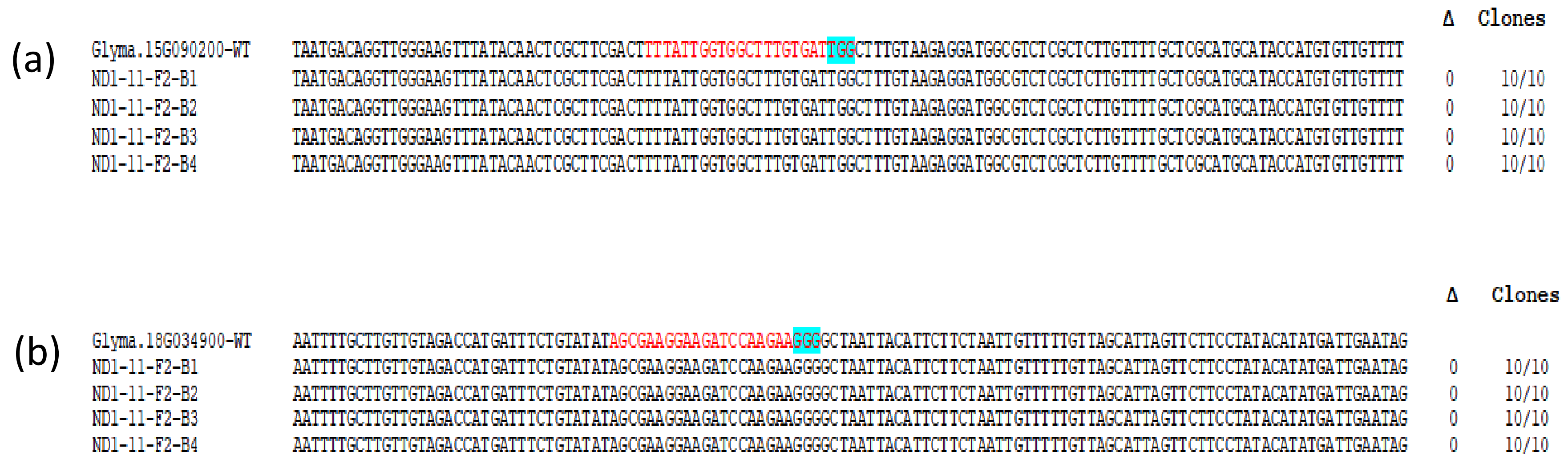


**Figure S5.** Sequencing results of off-target and flanking regions. a Off-target of gRNA1 (target 1) in chromosome15. b Off-target of gRNA2 (target 2) in chromosome18. Δ, Indels in target gene sequences (0: no deletion or insertion); Clones, number of mutated amplicons out of total sequenced clones.

**Table S1**. Primer sequences for genotyping *GmFAD2* gene mutations

| Primer pairs | Sequences | PCR products of WT | PCR products of mutants/Transgenic lines | Positions: Chromosome | Positions: Start |
|---|---|---|---|---|---|
| FAD2-1A-F | 5'-AGGCTTAGGTGTAGGCACCTAGC-3' | 1796 bp | ≤1796 bp | 10 | 50013799 |
| FAD2-1A-R | 5'- GTAATCCTCTAGATGAACCTCTGC-3' | | | | 50015594 |
| FAD2-1B-F | 5'-CTCTCTAATCTGTCACTTCCCTCC-3' | 1413 bp | ≤1413 bp | 20 | 35317536 |
| FAD2-1B-R | 5'-CACACAAGTCATTACGCGGC-3' | | | | 35318948 |
| D22-F | 5'-AGCCAGATGAAGGAACATCCG-3' | 396 bp | | 10 | 50015199 |
| FAD2-1A-R | 5'- GTAATCCTCTAGATGAACCTCTGC-3' | | | | 50015594 |
| D22-F | 5'-AGCCAGATGAAGGAACATCCG-3' | 162 bp | | 20 | 35318787 |
| FAD2-1B-R | 5'-CACACAAGTCATTACGCGGC-3' | | | | 35318948 |
| FAD2-1A-F | 5'-AGGCTTAGGTGTAGGCACCTAGC-3' | 407 bp | | 10 | 50013799 |
| D22-R | 5'-CAACAGTGAATGGTGGCTTTGT-3' | | | | 50014205 |
| FAD2-1B-F | 5'-CTCTCTAATCTGTCACTTCCCTCC-3' | 258 bp | | 20 | 35317536 |
| D22-R | 5'-CAACAGTGAATGGTGGCTTTGT-3' | | | | 35317793 |
| G18-off2-F2 | 5'-CGCATGATCATATTTGCTGGCT-3' | 245 bp | 245 bp | 18 | 2713153 |
| G18-off2-R | 5'-CTGATGCTGGTTCATAGCAGTG-3' | | | | 2713397 |
| G15-off1-F1 | 5'-GTGTCATGGTCCTGCACGTA-3' | 769 | 769 bp | 15 | 6943532 |
| G15-off1-R1 | 5'-GCAGAAGTGCTCCCATTCCT-3' | | | | 6942764 |
| Bar-F | 5'-TACCATGAGCCCAGAACGACGCCC-3' | 0 | 336 bp | - | - |
| Bar-R | 5'-CTTCAGCAGGTGGGTGTAGAGCG-3' | | | | |
| Cas-F | 5'-GCCCAAGAGGAACAGCGATAAGC-3' | 0 | 328 bp | - | - |
| Cas-R | 5'-CAGTTCGCCGGCAGAGGCCAGC-3' | | | | |
| F-FAD2-Crispr1 | 5'-GATTGATGAAGGAACATCCGAGAA-3' | - | - | - | - |
| R-FAD2-Crispr1 | 5'-AAACTTCTCGGATGTTCCTTCATC-3' | | | | |
| F-FAD2-cirspr2 | 5'-GATTGTGAATGGTGGCTTTGTGTT-3' | - | - | - | - |
| R-FAD2-Crispr2 | 5'-AAACAACACAAAGCCACCATTCAC-3' | | | | |

**Table S2.** Segregation of *Bar* and *Cas9* in T2 progenies derived from event ND1-11. Cas9 was detected by PCR while Bar was detected by both PCR and herbicide leaf painting.

| T2 lines | Bar -PCR | Leaf painting | Cas9-PCR | T2 lines | Bar -PCR | Leaf painting | Cas9-PCR |
|---|---|---|---|---|---|---|---|
| ND1-11-1-1 | + | R | + | ND1-11-2-1 | - | S | - |
| ND1-11-1-2 | + | R | + | ND1-11-2-2 | + | R | + |
| ND1-11-1-3 | + | R | + | ND1-11-2-3 | + | R | + |
| ND1-11-1-4 | + | R | + | ND1-11-2-4 | - | S | - |
| ND1-11-1-5 | + | R | + | ND1-11-2-5 | + | R | + |
| ND1-11-1-6 | + | R | + | ND1-11-2-6 | + | R | + |
| ND1-11-1-7 | + | R | + | ND1-11-2-7 | + | R | + |
| ND1-11-1-8 | + | R | + | ND1-11-2-8 | + | R | + |
| ND1-11-1-9 | + | R | + | ND1-11-2-9 | + | R | + |
| ND1-11-1-10 | + | R | + | ND1-11-2-10 | + | R | + |
| ND1-11-1-11 | + | R | + | ND1-11-2-11 | + | R | + |
| ND1-11-1-12 | + | R | + | ND1-11-2-12 | + | R | + |
| ND1-11-1-13 | + | R | + | ND1-11-2-13 | + | R | + |
| ND1-11-1-14 | + | R | + | ND1-11-2-14 | + | R | + |
| ND1-11-1-15 | + | R | + | ND1-11-2-15 | - | S | - |
| ND1-11-1-16 | + | R | + | ND1-11-2-16 | - | S | - |
| ND1-11-14-1 | - | S | - | ND1-11-2-17 | + | R | + |
| ND1-11-14-2 | - | S | - | ND1-11-2-18 | + | R | + |
| ND1-11-14-3 | - | S | - | ND1-11-2-19 | - | S | - |
| ND1-11-14-4 | - | S | - | ND1-11-2-20 | + | R | + |
| ND1-11-14-5 | - | S | - | ND1-11-10-1 | + | R | + |
| ND1-11-14-6 | - | S | - | ND1-11-10-2 | + | R | + |
| ND1-11-14-7 | - | S | - | ND1-11-10-3 | + | R | + |
| ND1-11-14-8 | - | S | - | ND1-11-10-4 | - | S | - |
| ND1-11-14-9 | - | S | - | ND1-11-10-5 | + | R | + |
| ND1-11-14-10 | - | S | - | ND1-11-10-6 | + | R | + |
| ND1-11-14-11 | - | S | - | ND1-11-10-7 | - | S | - |
| ND1-11-14-12 | - | S | - | ND1-11-10-8 | - | S | - |
| ND1-11-14-13 | - | S | - | ND1-11-10-9 | + | R | + |
| ND1-11-14-14 | - | S | - | ND1-11-10-10 | - | S | - |
| ND1-11-14-15 | - | S | - | | | | |
| ND1-11-14-16 | - | S | - | | | | |

+. Possitive; -. Negative; R. Resistance; S. Susceptible

**Table S3.** Potential off-target mutations in transgenic T2 plants derived from event ND1-11.

| Target sites | Potential off-targets | | | | No. of Bulks [b] | Identified mutant |
|---|---|---|---|---|---|---|
| | Sequences | MMs [a] | Gene locus | Region | | |
| FAD2-Target 1 | **T**T**T**A**T**TGGTGGCTTTGTG**A**TTGG | 4 | Glyma.15G090200 | exon | 4 | 0 |
| FAD2-Target 2 | AG**C**GAAGGAA**G**ATCC**A**AGAAGGG | 5 | Glyma.18G034900 | intron | 4 | 0 |

[a] Mismatched bases are shown in bold letters; [b] Each bulk contained equal DNA from 8-10 T2 plants

**Table S4**. Protein and oil content in ND1-11-14 and wild type (Williams 82 and Maverick) seeds. Measurements were performed over two years under greenhouse (2017) and field (2018) conditions.

| Environment (Year) | Seed samples | Protein Dry basis % | Oil Dry basis % | Linoleic acid Dry basis % | Oleic acid Dry basis % | Palmitic acid Dry basis % | Stearic acid Dry basis % |
|---|---|---|---|---|---|---|---|
| Greenhouse (2017) | ND1-11-1 | 37.8±1.44 [a] | 22.8±0.09 [a] | 7.49±0.51 [b] | 75.1±0.78 [a] | 7.93±0.44 [c] | 3.70±0.04 [b] |
| | Maverick | 32.8±1.45 [b] | 22.9±0.13 [a] | 61.4±0.93 [a] | 10.0±1.37 [b] | 11.1±0.27 [b] | 4.11±0.33 [ab] |
| | Williams 82 | 40.5±0.77 [a] | 19.9±0.57 [b] | 61.1±1.49 [a] | 7.79±1.95 [b] | 11.9±0.26 [a] | 4.32±0.12 [a] |
| Field (2018) | ND1-11-1-1 | 42.5±0.64 [a] | 21.3±0.20 [a] | 8.16±1.30 [c] | 71.9±1.67 [a] | 7.71±0.43 [b] | 3.95±0.19 [b] |
| | Maverick | 42.5±1.35 [a] | 20.9±0.71 [a] | 43.3±1.58 [b] | 29.3±2.31 [b] | 10.8±0.18 [a] | 4.46±0.09 [a] |
| | Williams 82 | 42.3±0.74 [a] | 20.9±0.21 [a] | 55.7±4.99 [a] | 15.6±6.46 [c] | 10.8±0.19 [a] | 4.18±0.20 [ab] |

Shown are means ± SD for n= 3. Different letters denote significant differences at $p<0.05$ using one-way ANOVA followed by a post-hoc Turkey's multiple range test.
